# Supplementary figures and images for: Knockout of IRF7 Highlights its Modulator Function of Host Response Against Avian Influenza Virus and the Involvement of MAPK and TOR Signaling Pathways in Chicken
Source: Genes (Basel). 2020 Apr 2;11(4):385. doi: 10.3390/genes11040385 (PMC7230310; doi:10.3390/genes11040385)

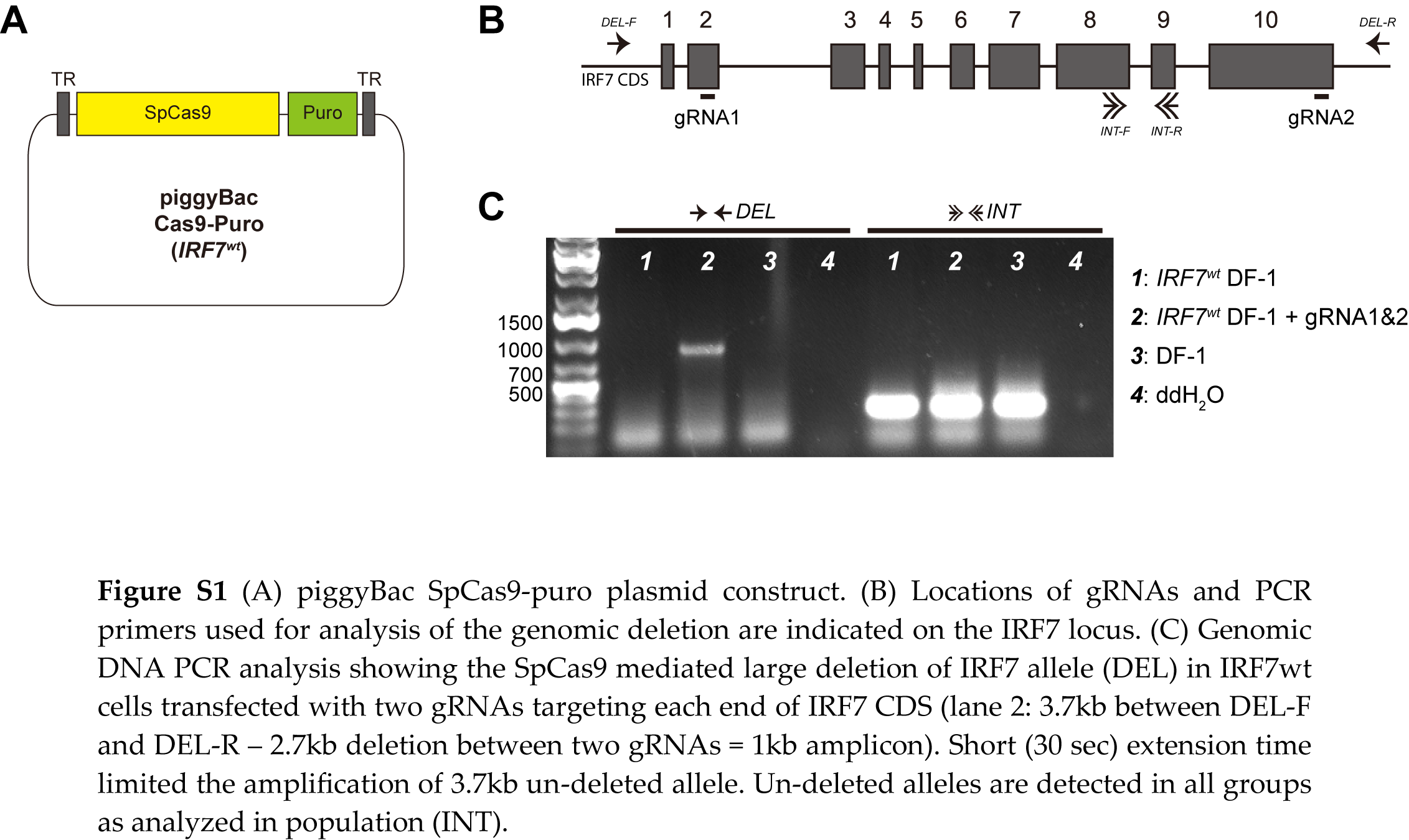

Supplement: Supplementary file 1 [file genes-11-00385-s001.zip › Figure_S1.tif]

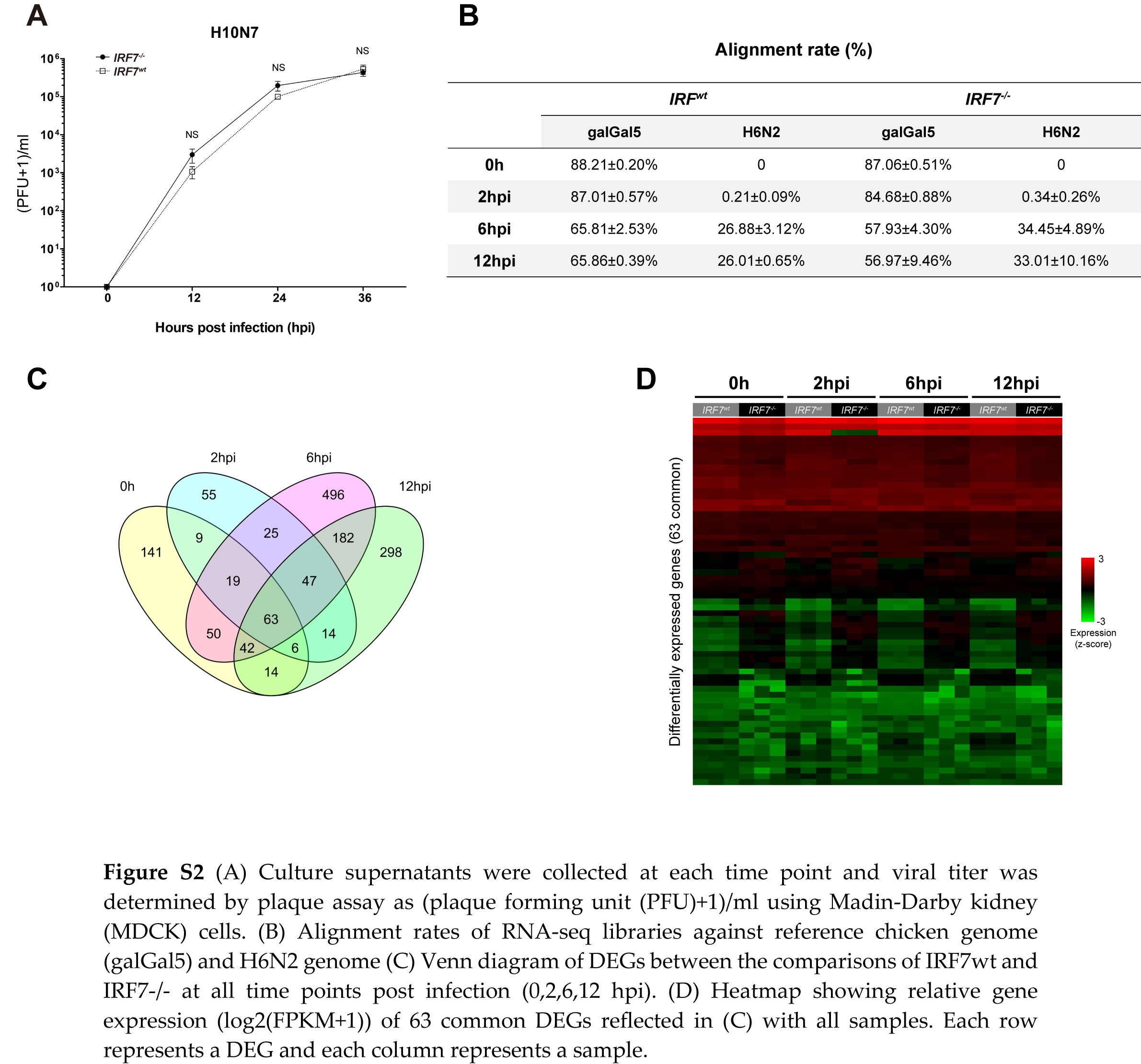

Supplement: Supplementary file 1 [file genes-11-00385-s001.zip › Figure_S2.tif]

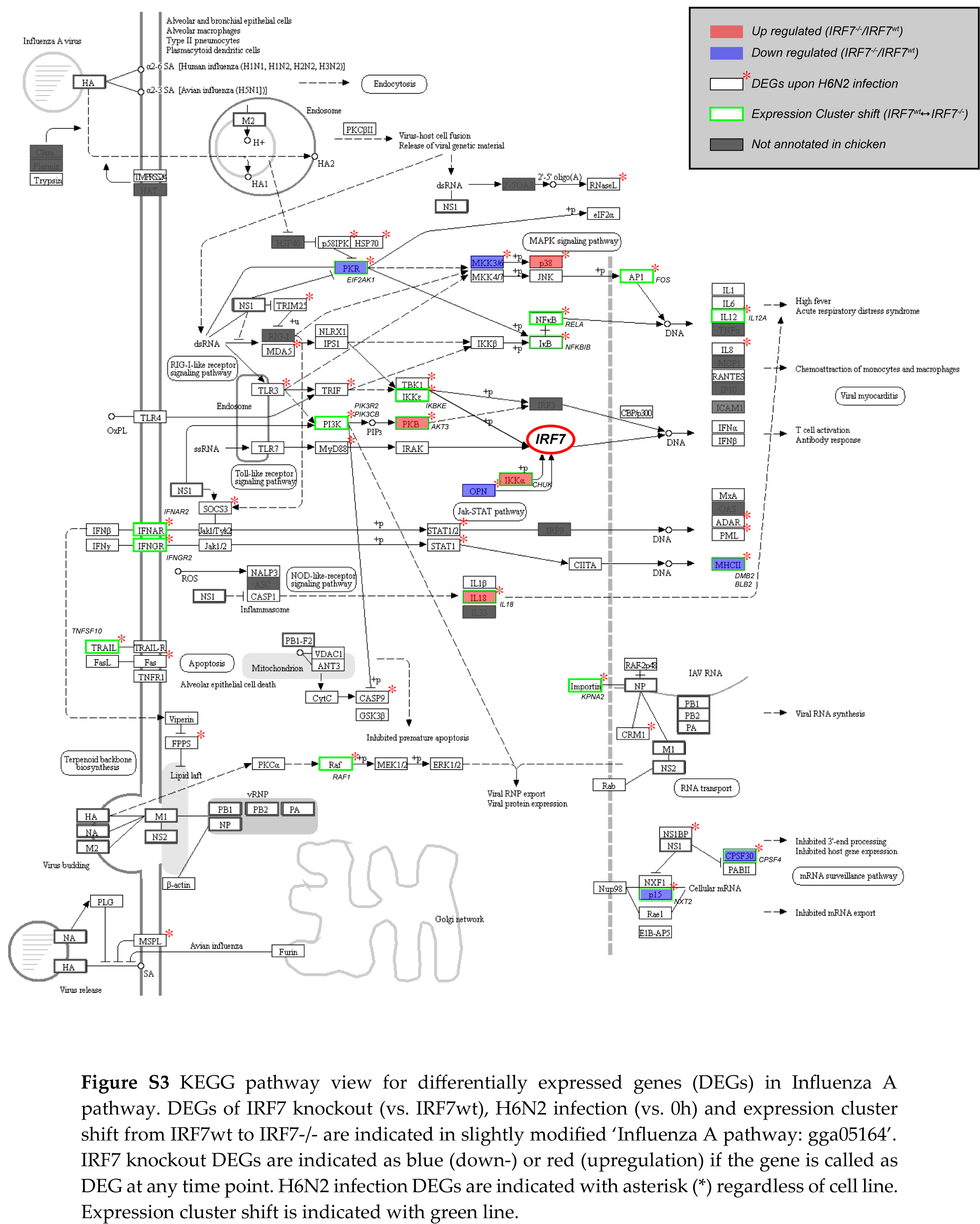

Supplement: Supplementary file 1 [file genes-11-00385-s001.zip › Figure_S3.tif]

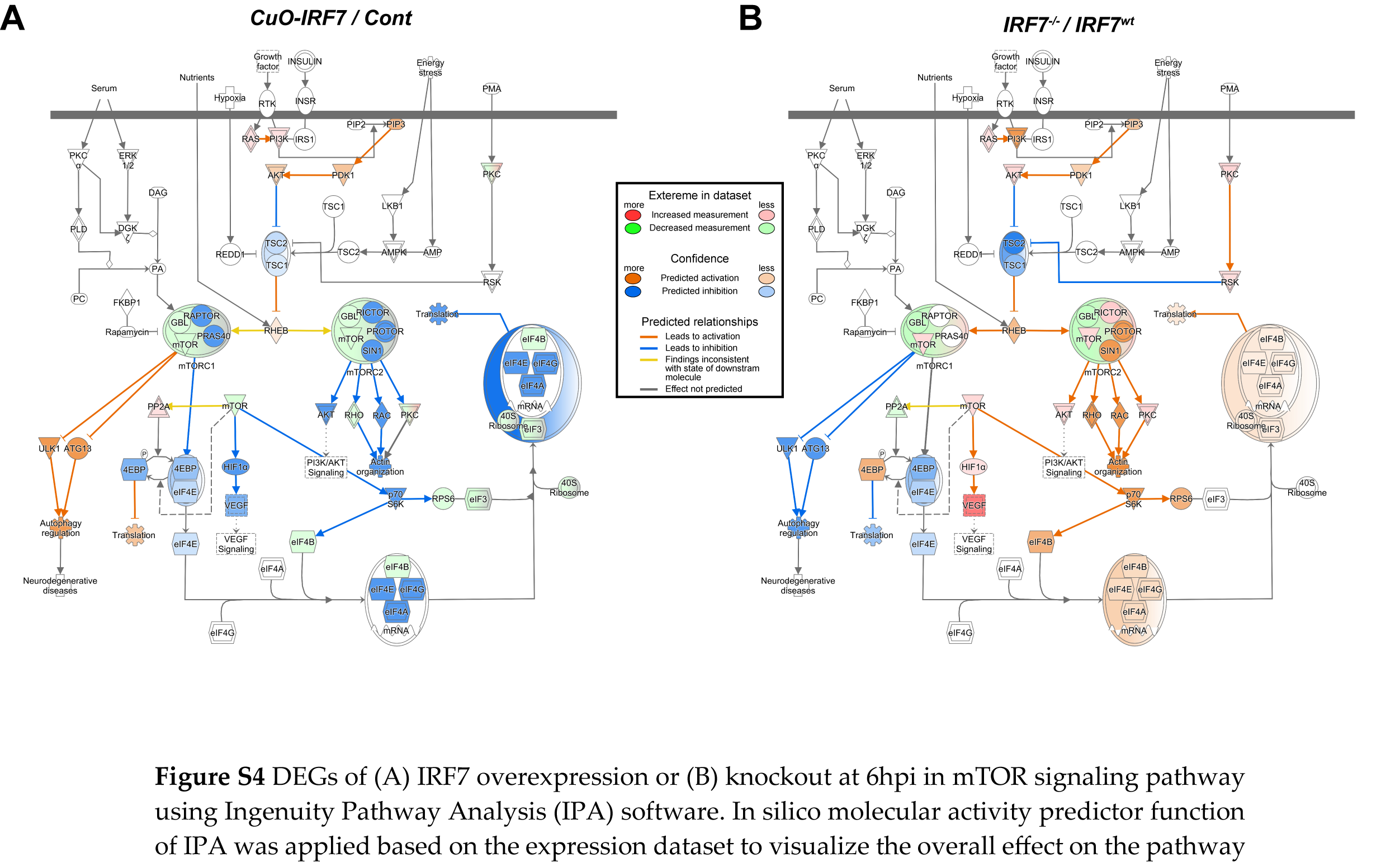

Supplement: Supplementary file 1 [file genes-11-00385-s001.zip › Figure_S4.tif]
